# Off to new shores: Climate niche expansion in invasive mosquitofish (*Gambusia* spp.)

**DOI:** 10.1002/ece3.8427

**Published:** 2021-12-13

**Authors:** Jonas Jourdan, Rüdiger Riesch, Sarah Cunze

**Affiliations:** ^1^ Department Aquatic Ecotoxicology Goethe University of Frankfurt Frankfurt am Main Germany; ^2^ Department of Biological Sciences Royal Holloway University of London Egham UK; ^3^ Department of Integrative Parasitology and Zoophysiology Goethe University of Frankfurt Frankfurt am Main Germany

**Keywords:** *Gambusia affinis*, *Gambusia holbrooki*, global climate change, invasion risk assessment, potential invasion area, species distribution model, invasive fish

## Abstract

**Aim:**

Formerly introduced for their presumed value in controlling mosquito‐borne diseases, the two mosquitofish *Gambusia affinis* and *G*. *holbrooki* (Poeciliidae) are now among the world's most widespread invasive alien species, negatively impacting aquatic ecosystems around the world. These inconspicuous freshwater fish are, once their presence is noticed, difficult to eradicate. It is, therefore, of utmost importance to assess their geographic potential and to identify their likely ability to persist under novel climatic conditions.

**Location:**

Global.

**Methods:**

We build species distribution models using occurrence data from the native and introduced distribution ranges to identify putative niche shifts and further ascertain the areas climatically suitable for the establishment and possible spread of mosquitofish.

**Results:**

We found significant niche expansions into climatic regions outside their natural climatic conditions, emphasizing the importance of integrating climatic niches of both native and invasive ranges into projections. In particular, there was a marked shift toward tropical regions in Asia and a clear niche shift of European *G*. *holbrooki*. This ecological flexibility partly explains the massive success of the two species, and substantially increases the risk for further range expansion. We also showed that the potential for additional expansion resulting from climate change is enormous—especially in Europe.

**Main conclusions:**

Despite the successful invasion history and ongoing range expansions, many countries still lack proper preventive measures. Thus, we urge policy makers to carefully evaluate the risk both mosquitofish pose to a particular area and to initiate appropriate management strategies.

## Graphical abstract

The study aimed to investigate the invasion success of one of the most widespread freshwater fish in the world: the closely related mosquitofish (*Gambusia affinis* and *G*. *holbrooki*). For this, we first explored potential niche shifts in the invaded regions and then provide risk assessments for future range expansion in the course of climate change.

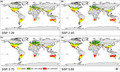

## 1 INTRODUCTION

Globalization with its massive global trade and long‐distance transportation is leading to a steady increase in the number of biological invasions, affecting all taxonomic groups and all continents, with no sign of saturation (Seebens et al., 2017). The rapidly changing climate is further facilitating the spread and establishment of invasive alien species (IAS; Hulme, 2017). IAS represent a major threat to biodiversity, challenging conservation efforts and management of biological resources (Simberloff et al., 2013). Accordingly, the Convention on Biological Diversity's (CBD) Strategic Plan for Biodiversity demanded a substantial increase in efforts made to reduce the impact and spread of invasive species, with prioritizing global actions of management and control (Essl et al., 2020; McGeoch et al., 2016). However, an essential prerequisite for the management and control of IAS is to understand the factors that determine the geographic distribution of a species and prevent it from spreading to other ecosystems.

The eastern mosquitofish, *Gambusia holbrooki*, and the closely related western mosquitofish, *G*. *affinis*, are one of the most successful freshwater IAS. They are native to the eastern and central United States, respectively, but have been introduced to every continent except Antarctica by aggressive introduction programs and their presumed value as mosquito control agents (Fryxell et al., in press; Krumholz, 1948; Pyke, 2008; Stockwell & Henkanaththegedara, 2011). Both species are tolerant toward anthropogenic disturbances (e.g., pesticides) and are capable of surviving a broad range of environmental conditions, as exemplified by tolerating salinities up to 41 ppt (Hubbs, 2000), temperatures between 0 and 40°C (Cherry et al., 1976; Lau et al., 2019), or oxygen contents ranging well into the hypoxic range (Cherry et al., 1976; Odum & Caldwell, 1955; Santi et al., 2020). These characteristics along with bearing live young contribute to their success as invasive species (Pyke, 2008; Walton et al., 2012). Collectively, *G*. *holbrooki* and *G*. *affinis* are among the most invasive fish worldwide and are currently considered as one of the 100 most detrimental IAS (Lowe et al., 2000). Their negative impact on local faunas stems partially from their often carnivorous feeding behavior (Pirroni et al., 2021; Pyke, 2008), and indigenous fish and amphibian larvae often rapidly decline after the introduction of mosquitofish (Barrier & Hicks, 1994; Morgan & Buttemer, 1996; Remon et al., 2016). Their dramatic effect for the local (often endemic) fauna has now been widely documented, especially in Australia (Arthington, 1989; Ivantsoff, 1999) and Europe (Alcaraz et al., 2008; Alcaraz & García‐Berthou, 2007; Carmona‐Catot et al., 2013; Rincon et al., 2002).

Despite this, *Gambusia* spp. together with other Poeciliid fishes (e.g., *Poecilia reticulata*) are still used as mosquito control agents in some parts of the world (Jayapriya & Shoba, 2014; Saleeza et al., 2014; Verma et al., 2016). From a conservation perspective it is, therefore, essential to identify regions where the—deliberate or accidental—introduction results in a high probability of establishment due to suitable (current and future) climatic conditions. Moreover, changing boundaries of already established populations also need to be robustly assessed. This is especially important, because prevention of spread (e.g., via control and public education) is more effective than trying to eradicate established populations (Fournier et al., 2019). A great tool for this type of assessment are ecological niche models (ENM; Guisan & Thuiller, 2005; Guisan & Zimmermann, 2000). ENMs are correlational techniques aimed to identify climatic regions where the species might find suitable conditions, based on current occurrence data and climatic information, and are broadly applied in the fields of biology, nature conservation, and biogeography (Elith et al., 2011). However, these models typically rely on the assumption that species retain their climatic niche in the exotic ranges (Peterson, 2011; Wiens et al., 2010). While this is true for many species, the full climatic potential is often not even fully realized due to sporadic introductions and a limited dispersal capacity (Pearson, 2007; Sillero, 2011). This is particularly pertinent for aquatic ecosystems where species mostly disperse within the river networks (Tonkin et al., 2018). Yet, some invasive species may even occur under climatic conditions that are outside the range of climatic values they inhabit within their native geographic distribution (Broennimann et al., 2007; Medley, 2010; Parravicini et al., 2015). Such a niche expansion can be facilitated by adaptive evolutionary processes in the novel distribution area (Reznick et al., 2019; Szűcs et al., 2017), different biotic interactions, or from preadaptation to conditions not available (anymore) in the species’ native range but available for the introduced populations (Guisan et al., 2014; Pearman et al., 2008). Hence, predicting future species distributions, by using only the native climatic niche, might severely underestimate the species’ geographic potential; this illustrates the importance of evaluating already existing niche shifts, and thus, the need to integrate non‐native occurrences when predicting the potential future range.

The exact global distribution of the two mosquitofish species has been difficult to establish for several reasons. First, both species are morphologically very similar and also hybridize, which makes identification challenging and results in many miss‐identifications (Scribner & Avise, 1993; Walters & Freeman, 2000). Second, both species were listed as a subspecies of *G*. *affinis* until 1988 (Pyke, 2008; Wooten et al., 1988). This complicates the evaluation of historical introduction events and results in a number of erroneous species identifications even today. For example, earlier literature reported the presence of *G*. *affinis* in Europe (Innal & Erk'akan, 2006; Krumholz, 1948), while more recent studies could only prove the presence of *G*. *holbrooki* (Santi et al., 2020; Sanz et al., 2013; Vidal et al., 2010). This is also the case for genetic studies of mosquitofish in Australia, where only *G*. *holbrooki* could be detected so far (Ayres et al., 2010, 2013). The situation is less clear for Asia, Latin America, and Africa, where we lack large‐scale genetic studies. However, regional molecular surveys suggest only one species, *G*. *affinis*, to be common in several southeast Asian countries (e.g., mainland China, Taiwan, Malaysia, Myanmar; Chang et al., 2019; Gao et al., 2017; Kano et al., 2016; Ouyang et al., 2018; Walton et al., 2016).

This global lack of clarity regarding the distribution of both species makes it challenging to evaluate the environmental requirements for both species separately. On the other hand, both species seem to pose a similar threat to indigenous fauna (Pyke, 2005, 2008), have a close taxonomic relationship (Lydeard et al., 1995), and similar ecologies (Walton et al., 2012). Moreover, there is an urgent need to identify the potential distribution range of these highly successful IAS as early as possible and thus establish strategies to detect and prevent further spread. In order to address these needs, our study sought to identify the areas climatically suitable for the establishment and possible spread of mosquitofish, while also considering potential niche shifts of established populations. More specifically—and in order to address different levels of certainty of species identification—we considered different taxonomic levels to address three interrelated questions:

1. In a first approach we treated both species together (i.e., combined species approach) and asked whether invasive mosquitofish conserve their climatic niche between native and introduced ranges. Therefore, we merged occurrence data from *G*. *affinis* and *G*. *holbrooki* and predicted that—due to an enormous introduction effort at the beginning of the last century—the global invasive range does already cover the entire climatic niche of the native range.
2. In a second, more speculative approach, we adopted a species‐specific approach, assuming that we can extrapolate evidence from genetic studies to all mosquitofish occurrences in the respective region. We predicted these models to reveal species‐specific climatic preferences that lead to different regional establishment probabilities for the two species.
3. Finally, we applied species distribution models to (a) identify areas prone to invasion under current climatic conditions and (b) to project climate change‐induced range shifts of both species. Such models will improve the assessments of species’ invasive potential and guide future management actions.

## 2 METHODS

We first examined and identified the climatic conditions under which the species occur (or have been successfully established) and compared these climatic niches between native range and non‐native range as well as between *G*. *affinis* and *G*. *holbrooki*. The climatic niche is defined (here) as the range of climatic conditions under which a species occurs (part of the niche space). Niche overlap indicates the range of climatic conditions under which two species can both occur; niche unfilling and niche expansion indicate the proportion where only one of the two species occurs, or—with regard to invasive species—niche unfilling refers to the climatic conditions under which the species occurs in the native range but not (yet) in the non‐native range. In analogy, niche expansion represents the range in which the species occurs in the non‐native range but not in the native range (Guisan et al., 2014).

Specifically, we compared (a) the native range niche with the non‐native range niche (combined species approach) and (b) the species‐specific niches by continent. Based on the niche comparisons using the framework implemented in the R package ecospat (see below), we then built an ecological niche model and projected the global climatic suitability for the two *Gambusia* species and thus the potential distribution of the species and estimate a future dispersal potential under changed future climate conditions.

### 2.1 Species distribution data

We obtained information on the global distribution for *G*. *affinis* and *G*. *holbrooki* from the Global Biodiversity Information Facility (GBIF, 2020), which covers information from several biodiversity databases including fishbase (Froese & Pauly, 2021). We further screened the existing literature to include additional records of *Gambusia affinis*/G. *holbrooki* establishment. From this, a total of 64,945 raw records were obtained for both species (Table A1). We used the R package CoordinateCleaner (Zizka et al., 2019) and the spThin package (Aiello‐Lammens et al., 2015) to flag potentially erroneous coordinates. Finally, a manual plausibility check was performed to validate the final dataset. We considered only one occurrence record per grid cell (spatial resolution of 10 arcmin as for climatic conditions) even if more than one occurrence was recorded, resulting in 8419 presences (4826 in the native range and 3593 in the non‐native ranges—combined species approach; and for the species‐specific approaches: for *G*. *affinis* 3745 native occurrences and 655 non‐native occurrences in North America, 139 occurrences in Asia, and for *G*. *holbrooki* 1290 native range occurrences in North America, 894 occurrences in Europe, and 1370 in Australia).

In a preliminary analysis we further cleaned the data based on the year of sampling so that they were in line with the climatic data (i.e., we only considered occurrences between 1970 and 2000). However, the spatial patterns in the occurrences vary between decades and do not reflect temporal changes in actual distribution but rather temporal changes in sampling effort. For example, almost all occurrences from the Iberian Peninsula are from the period after 2000, but it is well known that *G*. *holbrooki* was also widespread there in the period 1970–2000 (Krumholz, 1948; Pyke, 2008; Vidal et al., 2010). The massive and widespread introduction campaigns of *Gambusia* ended in the 1980s or earlier. Thus, the absence of the species in this area is due to a lack of sampling/reporting to GBIF and could not be explained by recent climatic changes. Accordingly, after careful consideration, we decided to continue working with the complete dataset, arguing that we would introduce a sampling bias into the data that is not justified by the advantage of matching the occurrence data to the related climate data.

**FIGURE A1:**
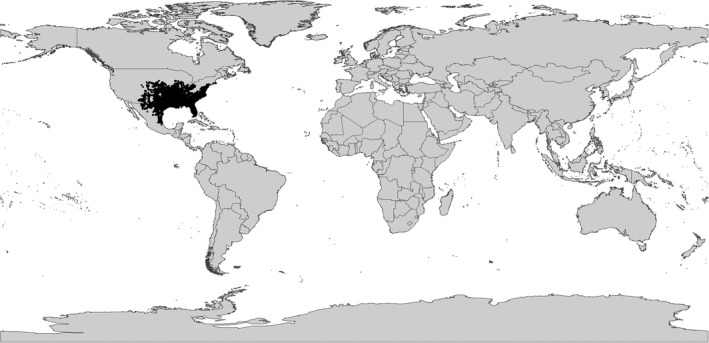
Occurrence records per grid cell for the combined native range of *Gambusia affinis and G*. *holbrooki* (Combined species approach; 11,622 data points)

**FIGURE A2:**
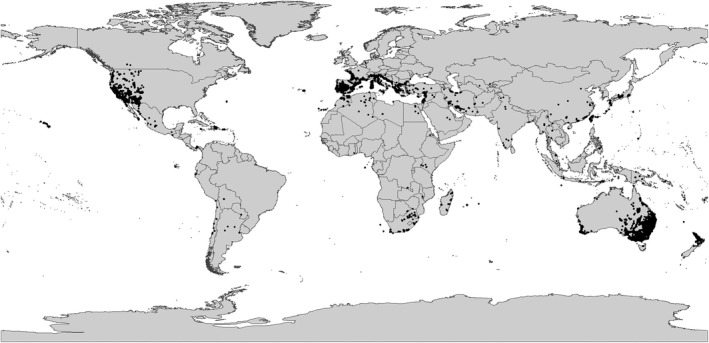
Occurrence records per grid cell for the combined invasive range of *Gambusia affinis and G*. *holbrooki* (Combined species approach; 6889 data points)

**FIGURE A3:**
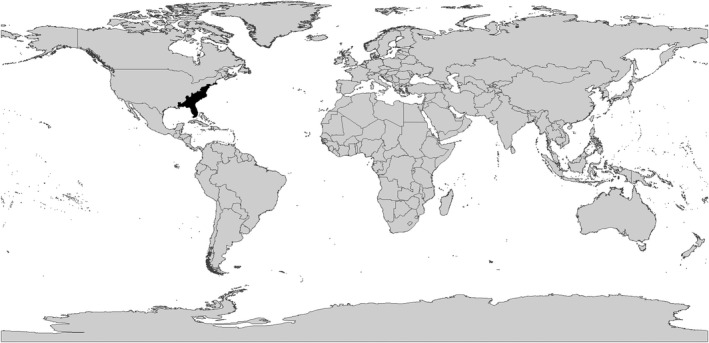
Occurrence records per grid cell for the native range of *Gambusia holbrooki* (3279 data points)

**FIGURE A4:**
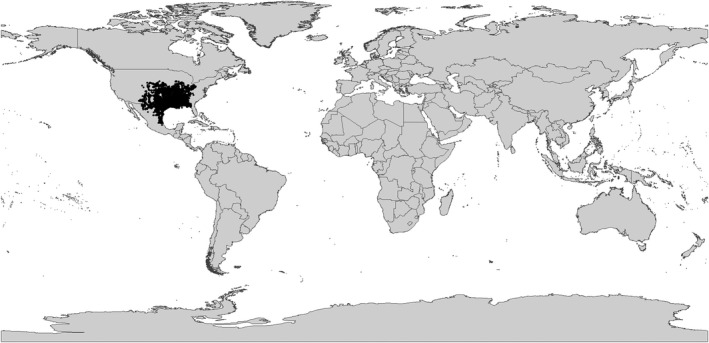
Occurrence records per grid cell for the native range of *Gambusia affinis* (8593 data points)

**FIGURE A5:**
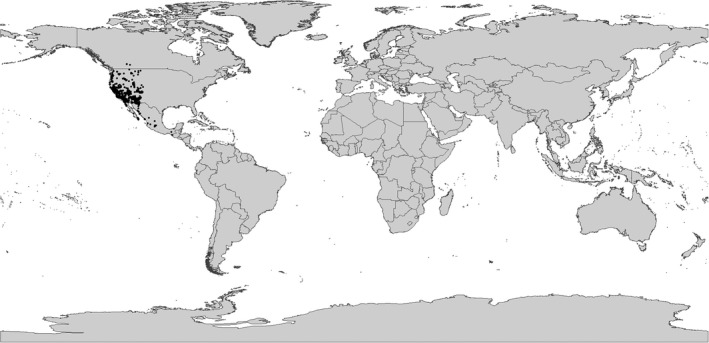
Occurrence records per grid cell for the invasive range of *Gambusia affinis* in North America (1167 data points)

**FIGURE A6:**
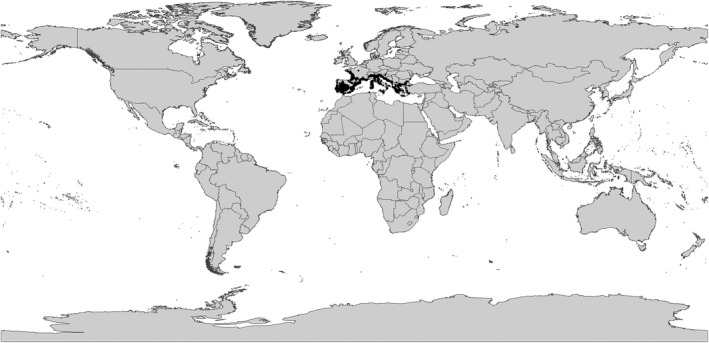
Occurrence records per grid cell for the invasive range of *Gambusia holbrooki* in Europe (1488 data points)

**FIGURE A7:**
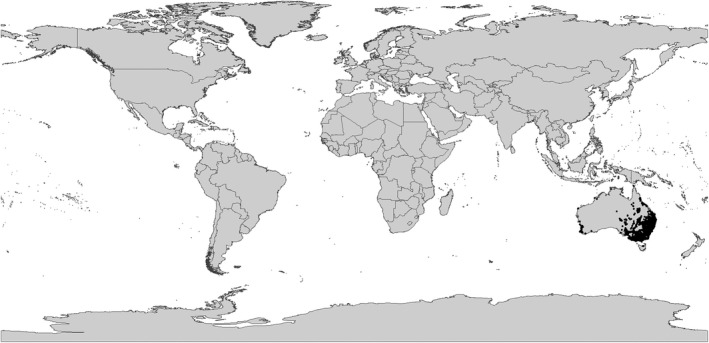
Occurrence records per grid cell for the invasive range of *Gambusia holbrooki* in Australia (3053 data points)

**FIGURE A8:**
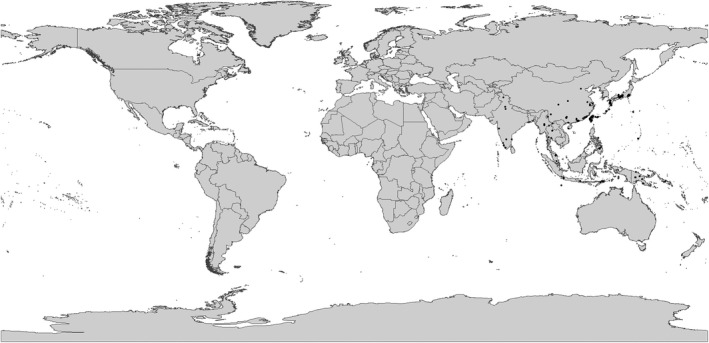
Occurrence records per grid cell for the invasive range of *Gambusia affinis* in southeast Asia (185 data points)

**FIGURE A9:**
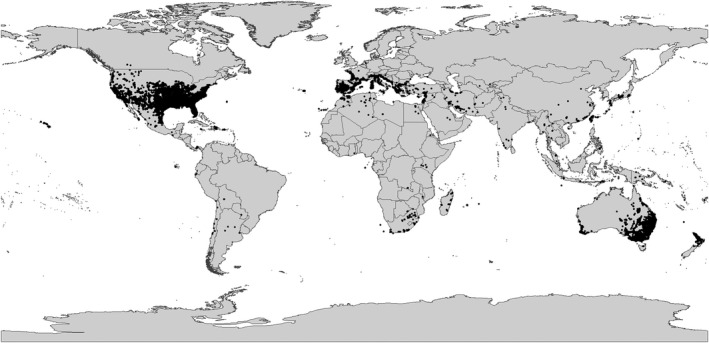
Global occurrence records per grid cell for *Gambusia affinis* and *G*. *holbrooki* (18,508 data points)

We defined species‐specific native ranges of both species according to recent genetic findings (Wilk & Horth, 2016), older references (Rauchenberger, 1989; Rosen & Bailey, 1963), and distribution information from the US Geological Survey (USGS, 2020). Occurrences that were north of the native distribution area but connected by river systems to the south were also classified as native, as we assumed that natural dispersal processes were just as likely as human introductions. Accordingly, the native range of *G*. *holbrooki* spans from Alabama, east into Florida, and north along coastal drainages to New Jersey (Figure A3), whereas *G*. *affinis* occurs from Alabama, west into New Mexico, and the Gulf drainages of eastern Mexico (Figure A4). A hybrid zone between the two closely related species can be found in the Mobile Bay, Alabama region (Wilk & Horth, 2016). In this area of overlapping distribution, we adopted the species information from the raw data (see Figures A1, A2, A3, A4, A5, A6, A7, A8, A9 for details).

### 2.2 Environmental variable selection

**FIGURE A10:**
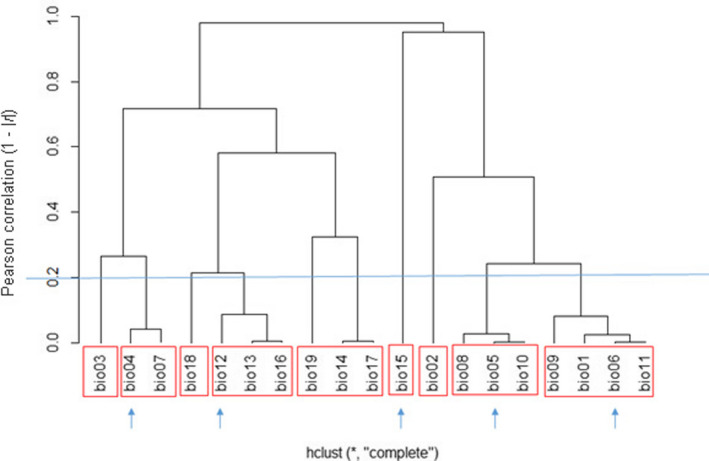
Cluster dendrogram with the Pearson's correlation coefficients (*r*) between the 19 climatic variables provided by worldclim. Based on a threshold of *r* ≥ |0.8 | (blue line) nine groups of intercorrelated variables were found. From five of these two groups we have chosen one representative that we consider to be ecologically most meaningful for the distribution of the fish species (blue arrows). We did not include additional precipitation variables (i.e., precipitation in the coldest [bio18, bio19] and the driest periods [bio14, bio17]), as well as the mean diurnal range (bio2) and isothermality (bio3), because we assume that diurnal differences in air temperature were thermally buffered by the water and, hence, less relevant for aquatic species

We used 19 climatic variables at 10‐arcminute resolution from the WorldClim database (Fick & Hijmans, 2017). To deal with strong collinearity among climatic variables, we calculated Pearson's correlation coefficients (*r*) between the 19 climatic variables and constructed a cluster dendrogram (Figure A10). Based on a threshold of *r* ≥ |0.8| (Elith et al., 2006; Franke, 2010; Mateo et al., 2013) nine groups of intercorrelated variables were found. From five of these groups, we have chosen one representative that we consider to be ecologically most meaningful for the distribution of the fish species (Gao et al., 2017; Riesch et al., 2018; Santi et al., 2020). The climatic variables that we selected for the analyses were temperature seasonality (bio4), maximum temperature of the warmest month (bio5), minimum temperature of the coldest month (bio6), annual precipitation (bio12), and precipitation seasonality (bio15). We did not include additional precipitation variables (i.e., precipitation in the coldest [bio19] and the driest periods [bio14, bio17]), as well as the mean diurnal range (bio2) and isothermality (bio3), because we assume that diurnal differences in air temperature were thermally buffered by the water and, hence, less relevant for aquatic species.

### 2.3 Comparison of the climatic niches

To quantify potential shifts in the niches of *G*. *affinis* and *G*. *holbrooki*, we used the Centroid, Overlap, Unfilling, Expansion (COUE) framework of Guisan et al. (2014) to decompose niche changes into centroid shifts, degree of overlap, and amounts of unfilling and expansion. We used the R package ecospat (Broennimann et al., 2020; Di Cola et al., 2017) to investigate the species’ distribution in the niche space. The resulting smoothed occurrence densities were plotted into the ordination space of PCA (based on five bioclimatic variables: bio04, bio05, bio06, bio12, bio15) to visualize the position of within environmental space (i.e., the realized/occupied climatic niche). Specifically, we compared native range and the non‐native range niches for both species together (combined species approach) and for each species separately (species‐specific approach). For each comparison of two niches, we calculated niche unfilling, defined as the percentage of the first niche covered by the second niche. In our case, a high unfilling means that a large part of the native range niche is unoccupied by the species in the non‐native range, or, when comparing two species, that there is a large range of environmental conditions under which the first species occurs but not the second. In addition, we calculated niche expansion, defined as the percentage of the second niche covered by the first niche. For our data, a high niche expansion means that the species occurs in the non‐native range under new conditions under which it does not occur in the native range, or, when comparing two species, that there is a large range of environmental conditions under which the second species occurs but not the first. Absolute overlap between the two given niches was further calculated based on the position of the occurrence densities using the *D* metric of Schoener (1968), which ranges between 0 (no overlap) and 1 (complete overlap).

### 2.4 Species distribution modeling

We projected the global habitat suitability for G. *affinis* and *G*. *holbrooki* (combined species approach) under current and future climatic conditions based on the ecological niche modeling approach. The ecological niche modeling was performed with an ensemble forecasting approach incorporating six state‐of‐the‐art niche modeling algorithms (ANN—artificial neuronal networks, GAM—generalized additive models, GBM—generalized boosted models, GLM—generalized linear models, FDA—flexible discriminant analysis, and RF—random forest) and executed in the R environment (R Development Core Team, 2019) using the biomod2 package (Thuiller et al., 2020). In the ensemble modeling approach, single model results are merged into a consensus model (here: weighted average), which is then considered to be a more robust estimator as it reduces uncertainties due to the choice of algorithm (Araújo & New, 2007). Applying an ensemble forecasting approach yields a robust projection of the species’ climate suitability (Araújo et al., 2005; Cunze et al., 2013).

In the ensemble model, we consider all presence–absence algorithms available in biomod2 (i.e., ANN, GAM, GBM, GLM, FDA, RF) and excluded the presence‐only algorithms (SRE) and presence background algorithms (Maxent), as we believe that due to the intensive introduction history, missing distribution data have a high information value and can be evaluated as absence data.

Ten thousand pseudo‐absences were chosen at random but excluding the area close to observed presences of the species in order to avoid pseudo‐replication, as close points tend to show similar environmental conditions/same niche (disk strategy implemented in the biomod2 package). The models were run using the following single algorithm parameters:

Running the artificial neuronal networks (ANN) we used five cross‐validation to find the best size and decay parameters, and set the initial random weights on [−0.1, 0.1] with 200 iterations at maximum. For generalized additive models (GAM) we used a binomial distribution and logit link function. Generalized boosted models (GBM) were run with a maximum of 2500 trees to ensure fitting, a minimum number of observations in trees’ terminal nodes of 10, a learning rate of 0.01, and an interaction depth of 7. To generate the generalized linear models (GLM), we applied a stepwise feature selection with quadratic terms based on the Akaike Information Criterion (AIC). RF was run with 500 trees and a node size of 5.

The performance (discriminatory capacity) of the algorithms was evaluated considering the receiver operating characteristic curve (ROC). A greater area under the curve (AUC) value indicates a better predictive model performance. For further performance measures, we applied the true skill statistic (TSS) which is a measure of predictive accuracy calculated based on the confusion matrix, that is, the TSS evaluates the binary model (in contrast to the AUC value which is a threshold independent measure). The TSS is defined as TSS = sensitivity + specificity—1 with sensitivity being the proportion of true positives (i.e., those positives modeled as positive) out of all positives in the evaluation dataset and specificity being the proportion of true negatives out of all negatives in the evaluation dataset. Consensus maps were built combining the modeling results of all algorithms with an AUC value >0.95. Their impact on the consensus maps was weighted by the mean of the AUC scores.

The ensemble model for current climatic conditions (Figure 3) successfully represents the current (combined) distribution described for *G*. *affinis* and *G*. *holbrooki*. Accordingly, we have adopted this as a baseline to study the potential distribution of both mosquitofish species under future conditions. We considered the future climate projections for the period 2081–2100, according to the fifth IPCC report (IPCC, 2014) and for four Shared Socio‐economic Pathways (SSPs), 1.26, 2.45, 3.70, and 5.85, processed based on the CNRM‐ESM2‐1 Global Circulation model (Seferian, 2018). Considering different SSPs, we account for different scenarios of future development based on different climate policies (SSP1: sustainability, SSP2: Middle paths, SSP3: Regional rivalry, SSP5: Fossil‐fueled development).

Continuous modeling results were transformed into binary results, using the equal sensitivity and specificity threshold (Liu et al., 2005). We subtracted the binary maps for the current conditions from the projected future distribution (2081–2100) to quantitate the impacts of climate change on range size changes. Based on these binary results (climatically suited or unsuited) under current and future climatic conditions, we thus identified areas that were (a) currently suited but projected to become climatically unsuitable in future (potential disappearance); (b) climatically suited under both current and future conditions (stable); and (c) currently unsuited but projected to become climatically suitable under future conditions (potential new range) (Figure 4). In order to make this information available for country‐specific management of invasive species, we provide a country‐specific invasion risk index. This index derived from the modeled habitat suitability averaged over all pixels of the respective country and was calculated for current and future climatic conditions (year 2081–2100; under four SSPs): minimal risk (0–14); low risk (0.15–0.24); moderate risk (0.25–0.49); and high risk (0.50–1.0).

We also looked at the variables’ contribution to the ensemble model of the considered five climate variables to identify important driving factors in the invasion process. In an analogous way, we have used the species‐specific occurrence data to model the species‐specific climatic suitability (Figures A18 and A19). Based on this (more speculative) approach, we identified areas that are climatically suited (according to our model) for both or only one of the two species, respectively (Figure A21).

Modeled habitat suitability was displayed in a map format created in ESRI ArcMap V10.8.1.

## 3 RESULTS

### 3.1 **Niche shift *Gambusia* spp**.

**FIGURE 1:**
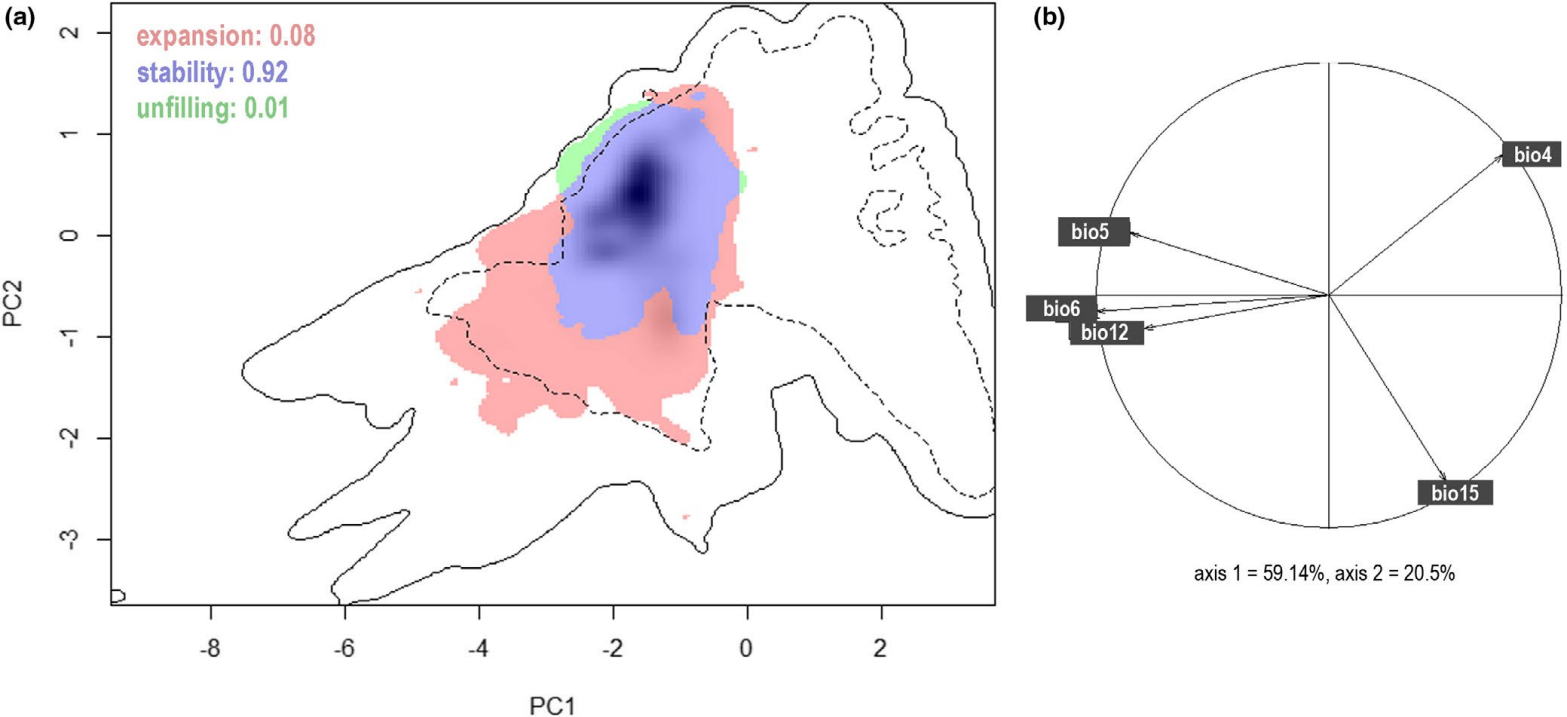
(a) Combined analysis of *Gambusia affinis* and *G*. *holbrooki* niche shift (combined species approach) in global environmental space, derived from principal component analysis on the climate predictors. Solid contour line represents available climates and dashed line the 50% most frequent available climate. Blue shaded area represents the niche area occupied in both native and invasive range; green area is the unfilled niche in the invasive range (relative to the native range), and pink shows the expansion area. The gray shading within these contours (black) correspond to the densities of occurrence records within the occupied climatic space of the latter niche (here: non‐native range niche). (b) Correlation circle indicates the weight of the selected climatic variables on the niche space as defined by the first two principal component axes (explaining 79.64% of the variance in the set of five predictor variables); bio4 = temperature seasonality, bio5 = maximum temperature of the warmest month, bio6 = minimum temperature of the coldest month, bio12 = annual precipitation, bio15 = precipitation seasonality

The niche shift analysis revealed that invasive mosquitofish have already exploited the full climatic range inhabited in their native range(s) (only 1.4% unfilling). Moreover, invasive mosquitofish expanded their niche by 8.4%, showing an expansion toward environments with higher precipitation seasonality (PS, dry, and rainy season) and into more tropical environments (higher TCM and AP; Figure 1a, b). This niche shift is also indicated by the Schoener's *D* index, which shows a low niche overlap between native *G*. *affinis*/G. *holbrooki* and their invasive ranges (*D* = 0.215; Table 1).

### 3.2 Species‐specific niche shifts

**FIGURE 2:**
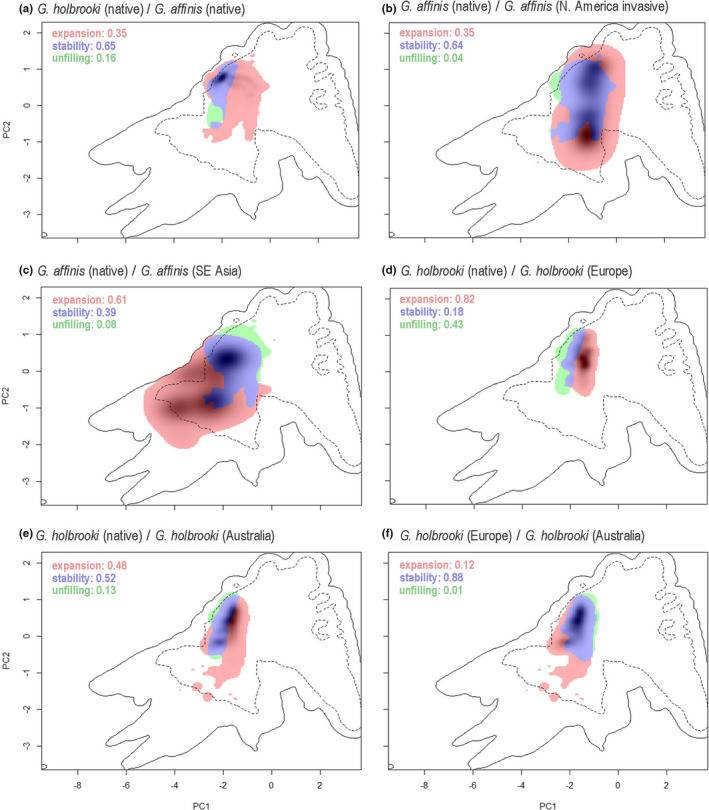
Projection of the realized niches in climatic space (species‐specific approach), comparing (a) populations within the native range of *Gambusia holbrooki* and *G*. *affinis*; native and invasive range of *G*. *affinis* in (b) North America and (c) Asia; native and invasive range of *G*. *holbrooki* in (d) Europe and (e) Australia, as well as (f) the two invasive ranges in Europe and Australia. Solid contour line represents available climates and dashed line the 50% most frequent available climate. Blue areas symbolize niche overlap; green area is the niche exclusively filled by the first‐mentioned species (i.e., “unfilled” by the second‐mentioned species), and pink shows the “expansion” area, that is, the climatic niche space solely occupied by the second‐mentioned species. The gray shading shows the smoothed occurrence density of the latter mentioned niche

Species‐specific niche comparisons provided further insights into continental distributions and niche shifts. Comparing the native niches of *G*. *affinis* and *G*. *holbrooki* revealed a high overlap of both species (niche stability = 65%, Schoener's *D* = 0.448; Table 1), but a generally larger niche space occupied by *G*. *affinis* (niche expansion = 35%; Figure 2a). Introduced populations of *G*. *affinis* at the West Coast of North America further expanded this niche space in North America by 35% (Figure 2b), because the invasive populations on the West Coast occur under very different climatic conditions than in the native range (Schoener's *D* = 0.080). We also found strong evidence for a pronounced expansion of *G*. *affinis’* realized niche in southeast Asia (60%; Figure 2c) toward more tropical conditions. Moreover, Schoener's *D* revealed an almost completely dislocated niche (*D* = 0.014) for Asian populations of *G*. *affinis*. Similarly, European populations of *G*. *holbrooki* showed a strong niche expansion (82%; Figure 2d) and occur in environments with greater seasonality (than in their native range)—representing climatic conditions more similar to those of *G*. *affinis* in their native range. Schoener's *D* revealed a low overlap (*D* = 0.187) of climatic niches between the native *G*. *holbrooki* range and the invasive range in Europe. In Australia, *G*. *holbrooki* colonized climatic conditions slightly more similar to those in their native range (niche stability = 52%; Schoener's *D* = 0.363), with additional occurrences in more tropical areas with higher precipitation seasonality (Figure 2e). The realized niche in Australia is similar to that in Europe (niche stability = 88%; Schoener's *D* = 0. 535; Figure 2f), but overall larger (niche expansion: 12%).

**TABLE 1:** Pairwise niche overlap indices (Schoener's *D*) of *Gambusia affinis* and *G*. *holbrooki* between native and invasive ranges

Approach	Combination	Schoener's *D*
Combined species approach	*G*. *affinis*/G. *holbrooki* native ‐ *G*. *affinis*/G. *holbrooki* invasive	0.215
Single‐species approach	*G*. *holbrooki* native ‐ *G*. *affinis* native	0.448
Single‐species approach	*G*. *affinis* native ‐ *G*. *affinis* invasive (North America)	0.080
Single‐species approach	*G*. *affinis* native ‐ *G*. *affinis* invasive (Asia)	0.014
Single‐species approach	*G*. *holbrooki* native ‐ *G*. *holbrooki* invasive (Europe)	0.187
Single‐species approach	*G*. *holbrooki* native ‐ *G*. *holbrooki* invasive (Australia)	0.363
Single‐species approach	*G*. *holbrooki* invasive (Europe) ‐ *G*. *holbrooki* invasive (Australia)	0.535

### 3.3 Projections of potential distributions

**FIGURE 3:**
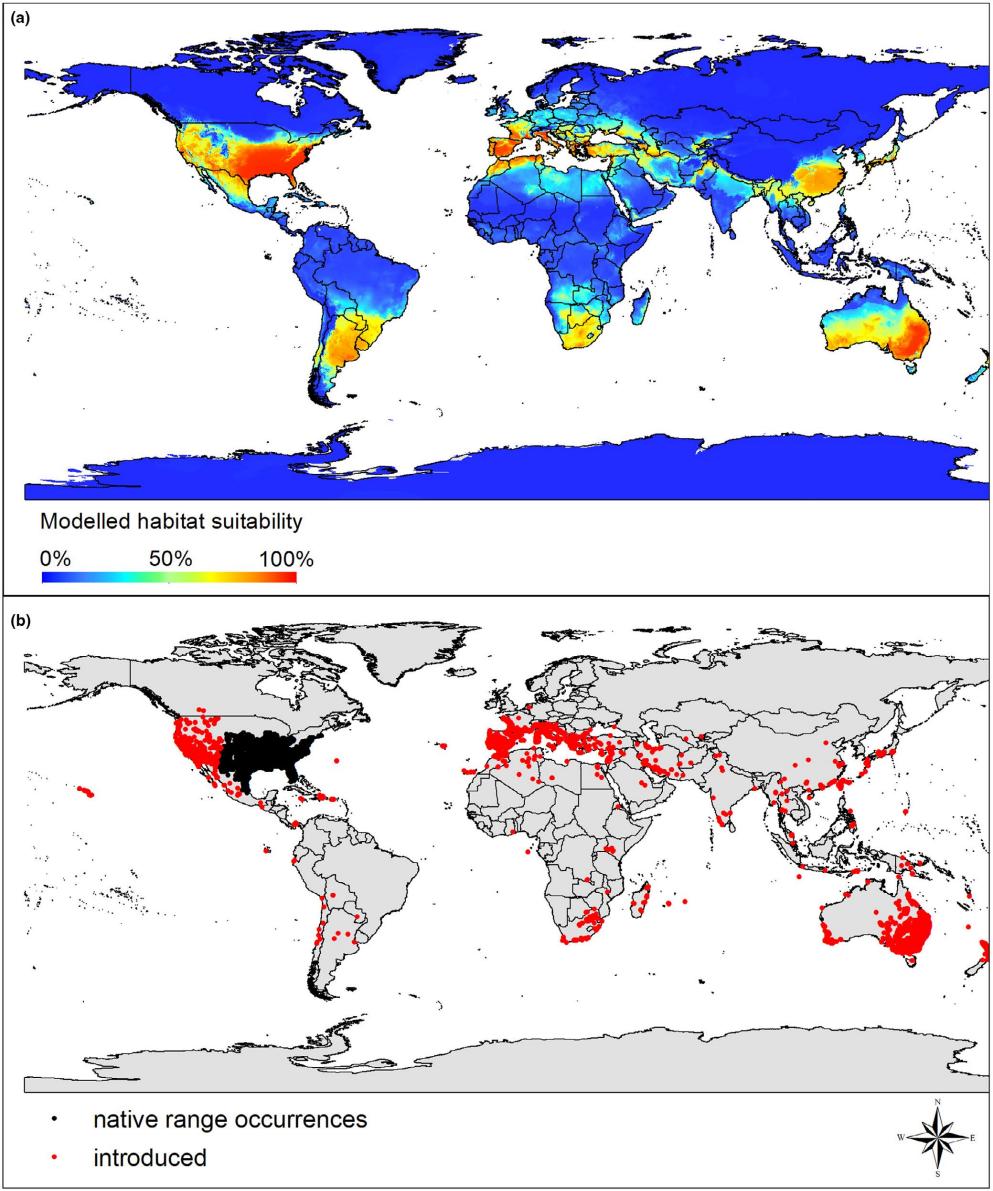
World map with (a) probability of the presence of *Gambusia affinis* or *G*. *holbrooki* predicted by the combined species approach build at the global scale using a consensus model with weighted (AUC) mean of six algorithms (GLM, GAM, GBM, ANN, FDA, RF). (b) The global distribution of known occurrences of *G*. *affinis* or *G*. *holbrooki*. Black dots indicate occurrences considered native in this study, and red dots represent occurrences considered as introduced

**FIGURE 4:**
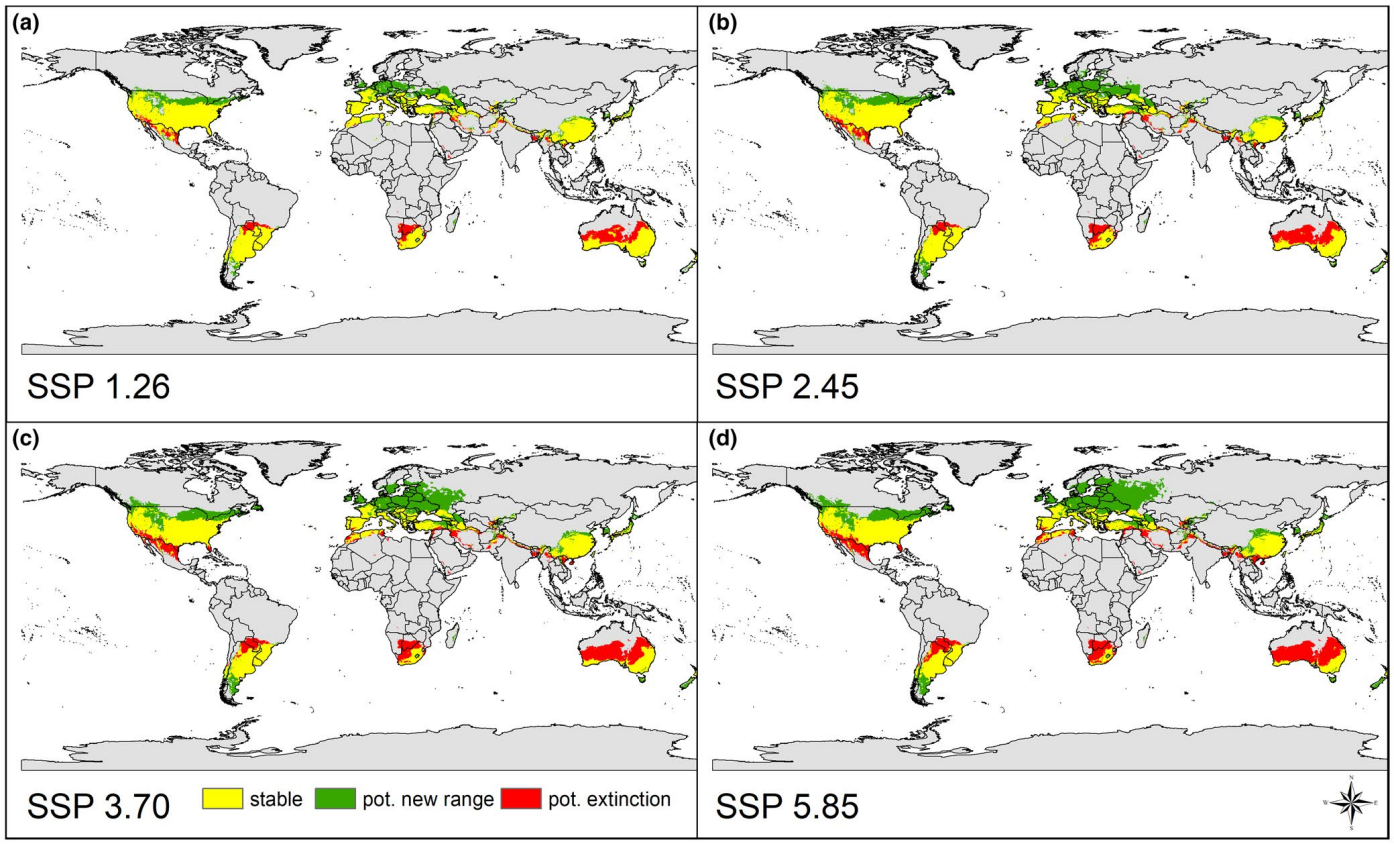
World map showing projected range shifts of *Gambusia affinis* and *G*. *holbrooki* until 2081–2100. These maps were derived from projected habitat suitability under current conditions relative to that under future (a) SSP 1.26, (b) SSP 2.45, (c) SSP 3.70, and (d) SSP 5.85 emission scenarios. Projections based on the binary (sensitivity equals specificity threshold) ensemble models (weighted [AUC] mean of six algorithms [GLM, GAM, GBM, ANN, FDA, RF]). Shown on the map are regions that become suitable (green), remain suitable (yellow), or become unsuitable (red) under future climate conditions

The current distribution of both mosquitofish species (combined species approach) covers large parts of the northern and southern hemisphere. Our modeling results support the observed distribution pattern under current climatic conditions (Figure 3), with AUCs  > 0.95 in the consensus model (for both mosquitofish species: TSS = 0.813, threshold = 26.65, sensitivity = 91.341, specificity = 89.940). However, when looking at the two species separately (species‐specific approach), small‐scale differences emerge. For example, *G*. *affinis* is more likely to find novel suitable conditions in western North America, while *G*. *holbrooki* is more likely to find novel suitable conditions in Central Europe (Figure 4; see also Figure A19, A20 for detailed species‐specific projections).

Under future climatic conditions (time period 2081–2100, Figure 4), the distribution ranges of both mosquitofish species (combined species approach) are predicted to expand. However, there are clear differences between the continents: While a northward range shift is predicted in North America and Europe, the opportunities for range expansion in the southern hemisphere are limited. Only in South America, a southern range expansion is to be expected. The greatest potential for range expansion can be expected in Europe. Regardless of the climate scenario considered, habitats in central Europe and southern United Kingdom will represent suitable habitats in the future. Assuming a moderate (SSP 3.70) or extreme (SSP 5.85) climate change scenario, the suitable habitat could even increase as far as southern Scandinavia and western Russia by the end of the century. In contrast, northern distributions in the southern hemisphere (e.g., parts of Australia, southern Botswana, southern Paraguay) become largely unsuitable for *G*. *holbrooki* and *G*. *affinis* under all models. The distribution range predictions were used to calculate a country‐specific assessment of the invasion risk (considering moderate to extreme climate change scenarios; see Appendix 7; Table A2).

## 4 DISCUSSION

In the first part of our paper, we explored niche dynamics of the highly invasive *G*. *affinis* and *G*. *holbrooki* in a combined species approach. In accordance with our associated prediction, we found that invasive mosquitofish occupy geographic areas that share the full range of climatic conditions occupied in the native range (only 1% unfilling). Moreover, our results suggest a slight expansion of the climatic niche by 8% during, or subsequent to, invasion of both species. This niche expansion becomes even more evident in our second, species‐specific approach: When comparing invasive populations of *G*. *holbrooki* in Europe with their native distribution range, we found a pronounced niche shift with a large degree of both expansion (81%) and low overlap with the native niche. Using a species‐specific approach for European populations is reasonable, because we have sufficient evidence from genetic studies that only *G*. *holbrooki* is present (Santi et al., 2020; Sanz et al., 2013; Vidal et al., 2010). The genetic evidence for the exclusive introduction of *G*. *holbrooki* is also good in Australia (Ayres et al., 2010, 2013), and here we also find a niche expansion, albeit less pronounced, compared to Europe (47% niche expansion). However, we found the most pronounced niche shift of invasive populations in Asia, where invasive populations have undergone almost a complete shift in their climatic niche. The classification of all Asian mosquitofish populations as G. affinis may be somewhat speculative (but see Chang et al., 2019; Gao et al., 2017; Kano et al., 2016; Ouyang et al., 2018; Walton et al., 2016); nonetheless, such tropical conditions are not inhabited by either species in the native range, providing impressive evidence of the species’ invasion potential and climatic flexibility.

Our species‐specific approach further outlines which components of the climatic niche have changed in different continents. In general, both species occupy slightly different native climatic niches, with *G*. *affinis* preferring a wider range of climatic conditions, while *G*. *holbrooki* prefers warmer regions with reduced seasonality. Interestingly, European populations of *G*. *holbrooki* changed their niche more toward climatic conditions similar to the native *G*. *affinis* niche (i.e., they occur under much colder conditions with higher seasonality than in the native *G*. *holbrooki* range). The massive shift of the climatic niche in Asian mosquitofish populations is mainly explained by the colonization of tropical regions with warm average temperatures and reduced seasonality. Knowledge on the spread of mosquitofish in the tropics, particularly in Asia, is very limited (Havel et al., 2015; Pyšek et al., 2008). In general, the observed expansion, especially in Asia, is not surprising due to the outstanding ability of both species to tolerate a wide range of environmental conditions, being extremely flexible in terms of their habitats (Pyke, 2008), diet (Pirroni et al., 2021) and abiotic conditions (Cherry et al., 1976; Odum & Caldwell, 1955). Mosquitofish respond to multiple interacting environmental factors by seemingly adaptive life‐history shifts (Riesch et al., 2018; Santi et al., 2020), which promote invasiveness and facilitate the colonization of new environments (Hendry, 2016). The observed niche expansion suggests that their realized niches in North America actually do not encompass their entire physiological and ecological ranges (Rosenfield, 2002). Indeed, native species’ distribution is often limited by biotic constraints (e.g., predation, competition, parasitism) and/or by biogeographical barriers (Moore et al., 2007; Richardson & Pyšek, 2008; Zaret, 1980). While the native distribution range of *G*. *holbrooki* is limited primarily by the Atlantic Ocean, the Gulf of Mexico, and/or competition from *G*. *affinis*, *G*. *affinis* encounters tremendous competition from other Poeciliidae at the southern range boundary, as Mexico already harbors >100 species of Poeciliidae, including 25 species of *Gambusia* (GBIF, 2021). If these constraints were removed, both mosquitofish species seem capable of occupying a much wider geographical and ecological range of habitats.

We detected these niche shifts even though we used a very conservative approach when defining the native range (i.e., we classified northern populations in eastern North America as native). Niche shifts following biological invasions have been repeatedly described (Broennimann et al., 2007; Gallagher et al., 2010), although a recent meta‐analysis for 434 invasive plant and animal species generally demonstrated that there is very limited niche expansion between native and introduced ranges (Liu et al., 2020). However, the occurrence of niche expansion was most evident in aquatic species, suggesting they might be more capable of invading diverse environments and highlighting the importance of aquatic habitats for conservation and species management (Liu et al., 2020). In the case of European mosquitofish, our study indicates that the niche shift occurred despite their low genetic diversity in Europe (Santi et al., 2020) and a short time span since the introduction at the beginning of the 20th century (Krumholz, 1948). We, therefore, can assume that the observed climatic niche shift of both mosquitofish might be a combination of microevolutionary change and/or adaptive plasticity, that is, shifts in the realized climatic niche within the broad fundamental niche of *G*. *affinis* and G. *holbrooki*. The latter matches recent findings regarding other aspects of their phenotype (Santi et al., 2020) and would represent a preadaptation to conditions that are not present in the native area (Cadotte et al., 2018; Petitpierre et al., 2012), indicating an intrinsic capacity to be successful invaders of novel environments.

Based on our finding that both mosquitofish expanded their realized niche globally beyond their native niche(s), we have described the potential range of *G*. *affinis* and *G*. *holbrooki* using the pooled occurrence data from native and invasive range. This represents a first step toward a risk analysis of both species, which are already recognized as highly problematic global invaders (Lowe et al., 2000). Our projections under future climatic scenarios are alarming and show that (particularly) large areas of Central Europe are predicted to become climatically suitable for mosquitofish in the future. So far, there have been no global models for the probability of establishment, and so it is not surprising that the risk of invasion is assessed differently by different European countries. For example, a recent horizon scan for potential future IAS threatening Great Britain biodiversity did not consider mosquitofish (Roy et al., 2014), while they are on the watch list in Germany, but without specific actions or plans for management (Nehring et al., 2010). The main reason for the severe risk of future mosquitofish establishment are elevated winter temperatures—which so far have prevented the establishment of *G*. *holbrooki* in central Europe (Kinzelbach & Krupp, 1982). This highlights the urgent need to devise appropriate species management plans for the areas and countries predicted to be affected in the near future.

A general shortcoming of species distribution models of aquatic species is that they often refer to terrestrial climate scenarios. Unfortunately, the resolution of the aquatic predictors (Domisch et al., 2015) is not yet sufficient to properly enable the analyses we conducted here. *Gambusia holbrooki* and *G*. *affinis* are species that occur in the smallest puddles and roadside ditches, about which no robust global environmental information is yet available. However, these small habitats are also strongly influenced by the surrounding air temperatures due to their low water masses/volumes, which is why we are convinced that the terrestrial climate data used here are suitable predictors.

**FIGURE A11:**
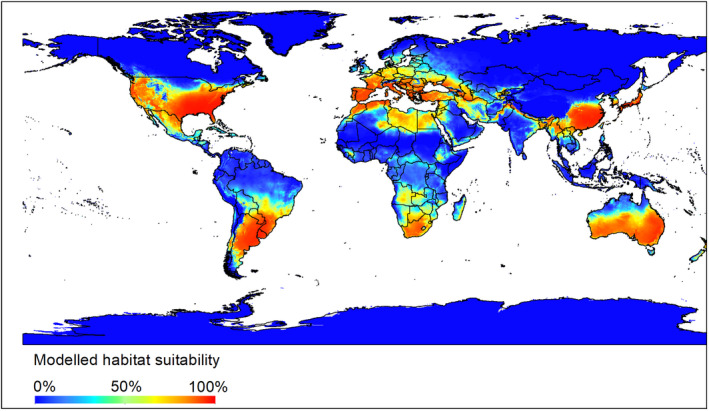
GLM model, both species together (Combined species approach)

**FIGURE A12:**
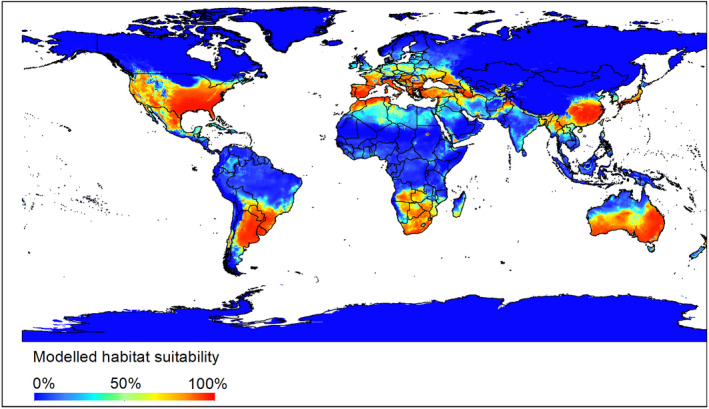
GAM model, both species together (Combined species approach)

**FIGURE A13:**
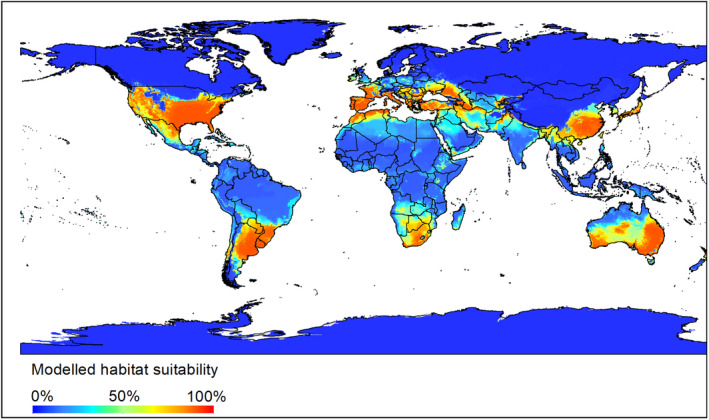
GBM model, both species together (Combined species approach)

**FIGURE A14:**
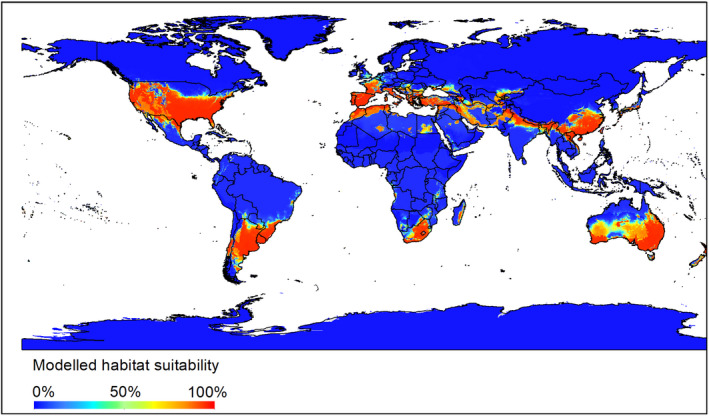
ANN model, both species together (Combined species approach)

**FIGURE A15:**
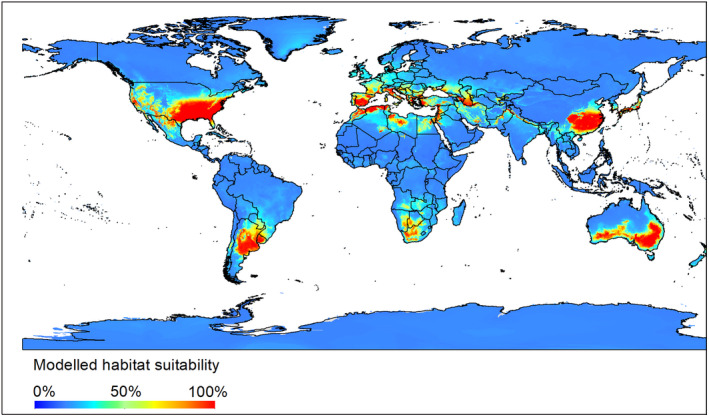
FDA model, both species together (Combined species approach)

**FIGURE A16:**
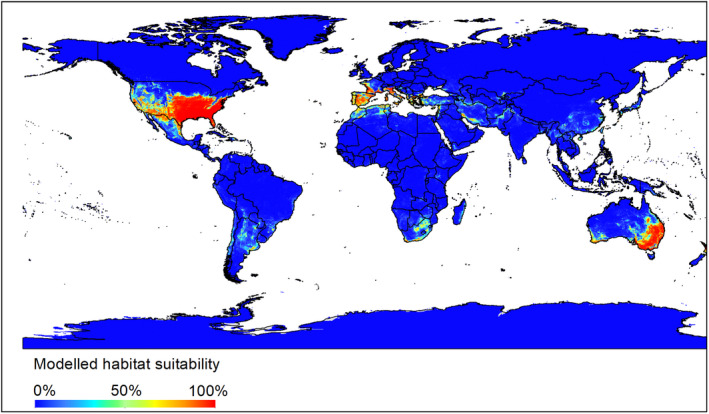
RF model, both species together (Combined species approach)

**FIGURE A17:**
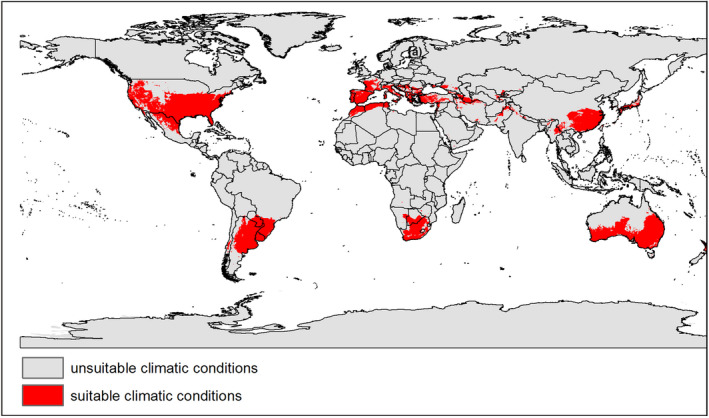
Projected climatic suitability (binary result applying the sensitivity equals specificity threshold) using global occurrence records of *Gambusia affinis* and *G*. *holbrooki* (combined species approach). Consensus model: weighted (AUC) mean of six algorithms (GLM, GAM, GBM, ANN, FDA, RF)

**FIGURE A18:**
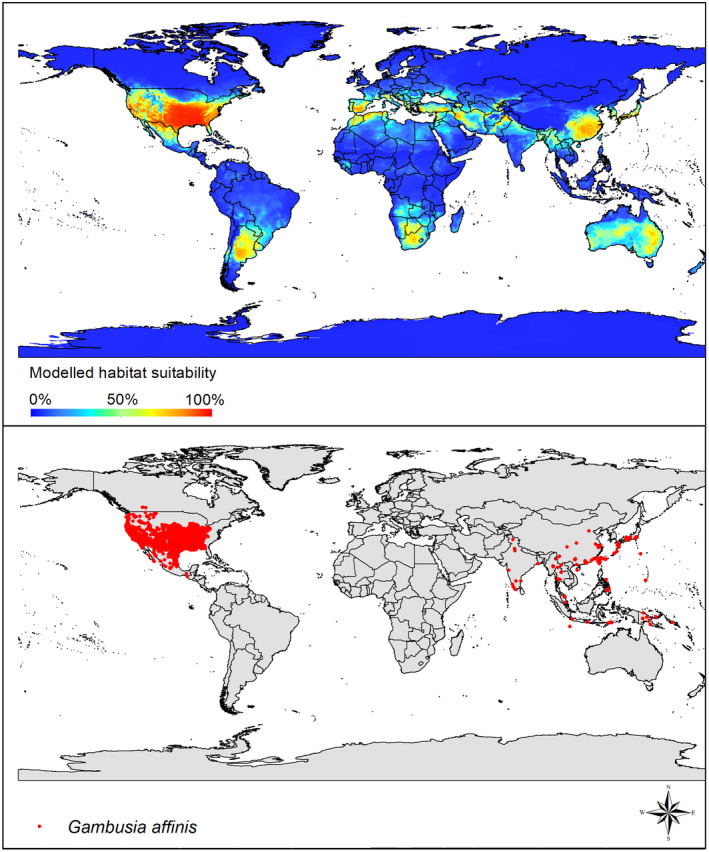
Projected climatic suitability (continuous results) using global occurrence records of *Gambusia affinis* (species‐specific approach). Consensus model: weighted (AUC) mean of six algorithms (GLM, GAM, GBM, ANN, FDA, RF)

**FIGURE A19:**
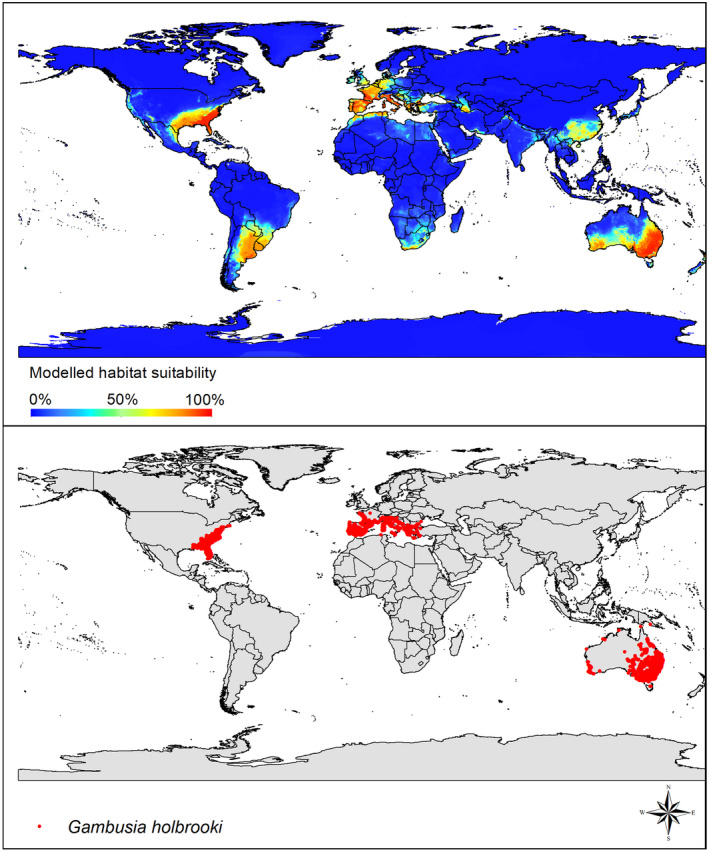
Projected climatic suitability (continuous results) using global occurrence records of *Gambusia holbrooki* (species‐specific approach). Consensus model: weighted (AUC) mean of six algorithms (GLM, GAM, GBM, ANN, FDA, RF)

**FIGURE A21:**
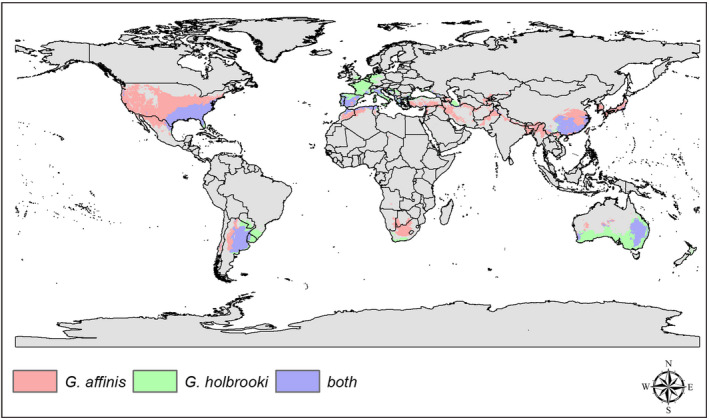
Projected current climatic suitability for *Gambusia affinis* (red), *Gambusia holbrooki* (green) or both species (purple). Projections based on the binary (sensitivity equals specificity threshold) species‐specific ensemble models (weighted [AUC] mean of six algorithms [GLM, GAM, GBM, ANN, FDA, RF], species‐specific approach)

In conclusion, our results show that a combination of niche conservatism and niche expansion facilitate a large climatic tolerance that helps explain the observed invasive success of both species in several parts of the world and indicate that there is potential for further range expansion in the face of global warming. The control and eradication of mosquitofish is often promoted by both governmental and scientific authorities (Fryxell et al., in press; Pyke, 2005, 2008). However, limited evidence exists on feasibility of mosquitofish removal from natural environments in which they are established but not native (Brookhouse & Coughran, 2010; Cano‐Rocabayera et al., 2019). Contingency plans should, therefore, focus on prevention, especially in regions with suitable climatic conditions. One important tool is to reduce anthropogenic pressures (e.g., habitat modification, dam construction, pollution loads) and put effort into the restoration of disturbed habitats, because it is becoming increasingly evident that IAS flourish primarily in anthropogenically disturbed river systems (Lee et al., 2017). (Table A3, Figures A11, A12, A13, A14, A15, A16, A17.)

## CONFLICT OF INTEREST

The authors have not declared any conflict of interest.

## AUTHOR CONTRIBUTIONS

**Jonas Jourdan:** Conceptualization (equal); Data curation (equal); Investigation (supporting); Project administration (equal); Visualization (supporting); Writing – original draft (lead); Writing – review & editing (equal). **Rüdiger Riesch:** Formal analysis (supporting); Investigation (supporting); Project administration (supporting); Writing – original draft (supporting); Writing – review & editing (equal). **Sarah Cunze:** Conceptualization (equal); Data curation (equal); Formal analysis (lead); Investigation (lead); Methodology (lead); Project administration (equal); Visualization (lead); Writing – original draft (supporting); Writing – review & editing (equal).

## Figures and Tables

**FIGURE A20:**
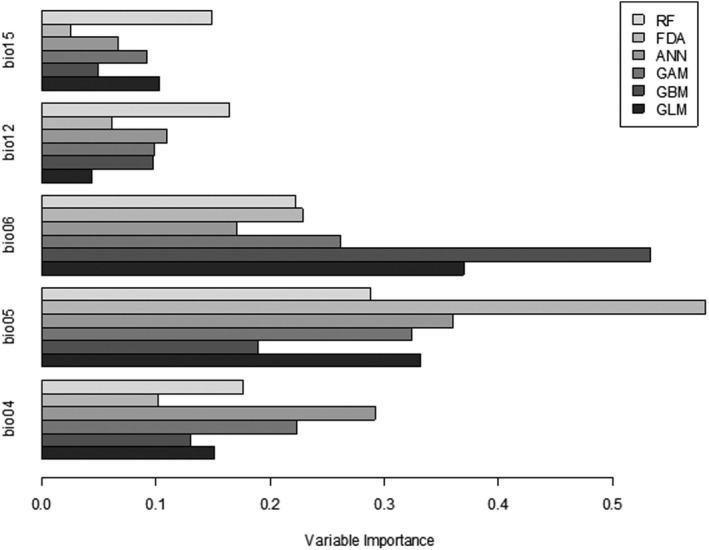
Variable importance of different models in the combined species approach

## Data Availability

The data that support the findings of this study are available in figshare at https://doi.org/10.6084/m9.figshare.14672286. All data used in this study are derived from public domain resources. The list of resources is given in Table A1.

## APPENDIX 1

### Species distribution data

**TABLE A1:** Data sources that provided distribution information of *Gambusia affinis*/*G*. *holbrooki* and were used for the analyses

Continent	Sources	Number of raw data points
All (global dataset)	Bayçelebi (2020), Cabral and Marques (1999), Cabrera et al. (2017), Gao et al. (2017), GBIF (2020), Kano et al. (2016), Landeka et al. (2015), Ouyang et al. (2018), Riesch et al. (2018), Santi et al. (2020), Walton et al. (2016), Wilk & Horth (2016), own unpublished data^a^	18,508
North America	GBIF (2020), Riesch et al. (2018), Wilk & Horth (2016)	12,789
Southeast Asia	Gao et al. (2017), GBIF (2020), Kano et al. (2016), Ouyang et al. (2018), Walton et al. (2016)	185
Europe	Cabral & Marques (1999), GBIF (2020), Landeka et al. (2015), Santi et al. (2020), own unpublished data^a^	1488
Australia	GBIF (2020)	3053

^a^ Includes a record from Sardinia, Italy (40.224845, 9.625957) and Lake Prespa, Greece (40.789083, 21.078232)

## APPENDIX 7

### Country‐specific invasion risk index for *G. affinis*/*G. holbrooki*

**TABLE A2:** Categorized establishment risk that at least one of the two species (*Gambusia affinis*/*G*. *holbrooki*) finds suitable climatic conditions in large parts of the respective country (under current and predicted climatic conditions of the 2081–2100 period; four SSPs). Relative climatic habitat suitability was calculated for each pixel in a country and averaged it over the total area of the country: minimal risk (0–14); low risk (0.15–0.24), moderate risk (0.25–0.49), and high risk (0.50–1.0). For large area countries that are climatically heterogeneous (e.g., China), this approach may result in low probabilities, although the probability of local establishment is very high. These countries are marked with an asterisk (*)

Country	Current	1.26 scenario	2.45 scenario	3.70 scenario	5.85 scenario
Afghanistan	Low risk	Moderate risk	Moderate risk	Moderate risk	Moderate risk
Albania	High risk	High risk	High risk	High risk	High risk
Algeria	Moderate risk	Moderate risk	Low risk	Low risk	Low risk
Andorra	Minimal risk	Moderate risk	Moderate risk	High risk	High risk
Angola	Low risk	Low risk	Low risk	Minimal risk	Minimal risk
Antarctica	Minimal risk	Minimal risk	Minimal risk	Minimal risk	Minimal risk
Antigua and Barbuda	Minimal risk	Minimal risk	Minimal risk	Minimal risk	Minimal risk
Argentina	High risk	High risk	High risk	High risk	High risk
Armenia	Low risk	High risk	High risk	High risk	High risk
Australia	High risk*	Moderate risk*	Moderate risk*	Moderate risk*	Moderate risk*
Austria	Low risk	Moderate risk	High risk	High risk	High risk
Azerbaijan	High risk	High risk	High risk	High risk	High risk
Bahamas	Moderate risk	Moderate risk	Moderate risk	Moderate risk	Moderate risk
Bahrain	Minimal risk	Minimal risk	Minimal risk	Minimal risk	Minimal risk
Bangladesh	Moderate risk	Moderate risk	Moderate risk	Low risk	Minimal risk
Barbados	Minimal risk	Minimal risk	Minimal risk	Minimal risk	Minimal risk
Belarus	Low risk	Moderate risk	Moderate risk	High risk	High risk
Belgium	Moderate risk	High risk	High risk	High risk	High risk
Belize	Low risk	Low risk	Low risk	Low risk	Low risk
Benin	Minimal risk	Minimal risk	Minimal risk	Minimal risk	Minimal risk
Bhutan	Low risk	Moderate risk	Moderate risk	Moderate risk	Moderate risk
Bolivia	Low risk	Low risk	Low risk	Low risk	Low risk
Bonaire	Minimal risk	Minimal risk	Minimal risk	Minimal risk	Minimal risk
Bosnia and Herzegovina	High risk	High risk	High risk	High risk	High risk
Botswana	High risk	Moderate risk	Low risk	Minimal risk	Minimal risk
Bouvet Island	Minimal risk	Minimal risk	Minimal risk	Minimal risk	Minimal risk
Brazil	Low risk*	Low risk*	Low risk*	Low risk*	Low risk*
British Virgin Islands	Low risk	Low risk	Low risk	Low risk	Minimal risk
Brunei Darussalam	Minimal risk	Minimal risk	Minimal risk	Minimal risk	Minimal risk
Bulgaria	High risk	High risk	High risk	High risk	High risk
Burkina Faso	Minimal risk	Minimal risk	Minimal risk	Minimal risk	Minimal risk
Burundi	Minimal risk	Minimal risk	Minimal risk	Minimal risk	Minimal risk
Cabo Verde	Minimal risk	Minimal risk	Minimal risk	Minimal risk	Minimal risk
Cambodia	Minimal risk	Minimal risk	Minimal risk	Minimal risk	Minimal risk
Cameroon	Minimal risk	Minimal risk	Minimal risk	Minimal risk	Minimal risk
Canada	Minimal risk	Minimal risk	Minimal risk	Minimal risk	Low risk
Canarias	Low risk	Moderate risk	Moderate risk	Moderate risk	Moderate risk
Central African Republic	Minimal risk	Minimal risk	Minimal risk	Minimal risk	Minimal risk
Chad	Minimal risk	Minimal risk	Minimal risk	Minimal risk	Minimal risk
Chile	Low risk	Low risk	Moderate risk	Moderate risk	Moderate risk
China	Low risk*	Low risk*	Moderate risk*	Moderate risk*	Moderate risk*
Christmas Island	Minimal risk	Low risk	Low risk	Low risk	Minimal risk
Colombia	Minimal risk	Minimal risk	Minimal risk	Minimal risk	Minimal risk
Comoros	Low risk	Low risk	Low risk	Low risk	Low risk
Congo	Minimal risk	Minimal risk	Minimal risk	Minimal risk	Minimal risk
Congo DRC	Minimal risk	Minimal risk	Minimal risk	Minimal risk	Minimal risk
Cook Islands	Moderate risk	Moderate risk	Moderate risk	Moderate risk	Moderate risk
Costa Rica	Minimal risk	Minimal risk	Minimal risk	Minimal risk	Minimal risk
Côte d'Ivoire	Minimal risk	Minimal risk	Minimal risk	Minimal risk	Minimal risk
Croatia	High risk	High risk	High risk	High risk	High risk
Cuba	Low risk	Low risk	Low risk	Low risk	Low risk
Curacao	Minimal risk	Minimal risk	Minimal risk	Minimal risk	Minimal risk
Cyprus	High risk	High risk	High risk	Moderate risk	Moderate risk
Czech Republic	Moderate risk	High risk	High risk	High risk	High risk
Denmark	Minimal risk	Moderate risk	Moderate risk	High risk	High risk
Djibouti	Minimal risk	Minimal risk	Minimal risk	Minimal risk	Minimal risk
Dominica	Minimal risk	Low risk	Minimal risk	Low risk	Minimal risk
Dominican Republic	Low risk	Low risk	Low risk	Low risk	Low risk
Ecuador	Minimal risk	Minimal risk	Minimal risk	Minimal risk	Minimal risk
Egypt	Low risk	Low risk	Low risk	Minimal risk	Minimal risk
El Salvador	Minimal risk	Minimal risk	Minimal risk	Minimal risk	Minimal risk
Equatorial Guinea	Minimal risk	Minimal risk	Minimal risk	Minimal risk	Minimal risk
Eritrea	Minimal risk	Minimal risk	Minimal risk	Minimal risk	Minimal risk
Estonia	Low risk	Moderate risk	Moderate risk	High risk	High risk
Eswatini	Moderate risk	Moderate risk	Moderate risk	Moderate risk	Moderate risk
Ethiopia	Minimal risk	Minimal risk	Minimal risk	Minimal risk	Minimal risk
Falkland Islands	Minimal risk	Minimal risk	Minimal risk	Low risk	Low risk
Faroe Islands	Minimal risk	Low risk	Low risk	Low risk	Low risk
Fiji	Low risk	Moderate risk	Moderate risk	Low risk	Low risk
Finland	Minimal risk	Low risk	Moderate risk	Moderate risk	Moderate risk
France	High risk	High risk	High risk	High risk	High risk
French Guiana	Minimal risk	Minimal risk	Minimal risk	Minimal risk	Minimal risk
French Polynesia	Low risk	Low risk	Low risk	Low risk	Low risk
French Southern Territories	Minimal risk	Minimal risk	Minimal risk	Minimal risk	Minimal risk
Gabon	Minimal risk	Minimal risk	Minimal risk	Minimal risk	Minimal risk
Gambia	Minimal risk	Minimal risk	Minimal risk	Minimal risk	Minimal risk
Georgia	Moderate risk	High risk	High risk	High risk	High risk
Germany	Moderate risk	High risk	High risk	High risk	High risk
Ghana	Minimal risk	Minimal risk	Minimal risk	Minimal risk	Minimal risk
Greece	High risk	High risk	High risk	High risk	High risk
Greenland	Minimal risk	Minimal risk	Minimal risk	Minimal risk	Minimal risk
Grenada	Minimal risk	Minimal risk	Minimal risk	Minimal risk	Minimal risk
Guadeloupe	Low risk	Low risk	Low risk	Low risk	Low risk
Guam	Low risk	Minimal risk	Minimal risk	Minimal risk	Minimal risk
Guatemala	Low risk	Low risk	Low risk	Minimal risk	Minimal risk
Guinea	Minimal risk	Minimal risk	Minimal risk	Minimal risk	Minimal risk
Guinea‐Bissau	Minimal risk	Minimal risk	Minimal risk	Minimal risk	Minimal risk
Guyana	Minimal risk	Minimal risk	Minimal risk	Minimal risk	Minimal risk
Haiti	Low risk	Low risk	Low risk	Minimal risk	Minimal risk
Heard Island and McDonald Islands	Minimal risk	Minimal risk	Minimal risk	Minimal risk	Minimal risk
Honduras	Low risk	Low risk	Low risk	Minimal risk	Minimal risk
Hungary	High risk	High risk	High risk	High risk	High risk
Iceland	Minimal risk	Minimal risk	Minimal risk	Minimal risk	Minimal risk
India	Low risk*	Minimal risk*	Minimal risk*	Minimal risk*	Minimal risk*
Indonesia	Minimal risk	Minimal risk	Minimal risk	Minimal risk	Minimal risk
Iran	Moderate risk	Moderate risk	Moderate risk	Moderate risk	Moderate risk
Iraq	Low risk	Moderate risk	Low risk	Low risk	Low risk
Ireland	Low risk	Moderate risk	High risk	High risk	High risk
Isle of Man	Low risk	Moderate risk	Moderate risk	High risk	High risk
Israel	Moderate risk	Moderate risk	Moderate risk	Low risk	Low risk
Italy	High risk	High risk	High risk	High risk	High risk
Jamaica	Minimal risk	Minimal risk	Minimal risk	Minimal risk	Minimal risk
Japan	High risk	High risk	High risk	High risk	High risk
Jersey	Moderate risk	High risk	High risk	High risk	High risk
Jordan	Moderate risk	Moderate risk	Moderate risk	Moderate risk	Low risk
Kazakhstan	Minimal risk*	Low risk*	Low risk*	Moderate risk*	Moderate risk*
Kenya	Minimal risk	Minimal risk	Minimal risk	Minimal risk	Minimal risk
Kiribati	Minimal risk	Minimal risk	Minimal risk	Minimal risk	Minimal risk
Kuwait	Low risk	Minimal risk	Minimal risk	Minimal risk	Minimal risk
Kyrgyzstan	Minimal risk	Low risk	Moderate risk	Moderate risk	Moderate risk
Laos	Moderate risk	Moderate risk	Moderate risk	Moderate risk	Low risk
Latvia	Low risk	Moderate risk	Moderate risk	High risk	High risk
Lebanon	High risk	High risk	High risk	High risk	High risk
Lesotho	Moderate risk	High risk	High risk	High risk	High risk
Liberia	Minimal risk	Minimal risk	Minimal risk	Minimal risk	Minimal risk
Libya	Moderate risk	Moderate risk	Low risk	Minimal risk	Minimal risk
Liechtenstein	Minimal risk	Low risk	Moderate risk	High risk	High risk
Lithuania	Low risk	Moderate risk	High risk	High risk	High risk
Luxembourg	Moderate risk	High risk	High risk	High risk	High risk
Madagascar	Low risk	Low risk	Low risk	Low risk	Low risk
Malawi	Low risk	Low risk	Minimal risk	Minimal risk	Minimal risk
Malaysia	Minimal risk	Minimal risk	Minimal risk	Minimal risk	Minimal risk
Mali	Minimal risk	Minimal risk	Minimal risk	Minimal risk	Minimal risk
Malta	High risk	High risk	High risk	Moderate risk	Moderate risk
Martinique	Minimal risk	Minimal risk	Minimal risk	Minimal risk	Minimal risk
Mauritania	Minimal risk	Minimal risk	Minimal risk	Minimal risk	Minimal risk
Mauritius	Moderate risk	Moderate risk	Moderate risk	Moderate risk	Moderate risk
Mayotte	Minimal risk	Minimal risk	Minimal risk	Minimal risk	Minimal risk
Mexico	Moderate risk	Moderate risk	Moderate risk	Moderate risk	Low risk
Micronesia	Minimal risk	Minimal risk	Minimal risk	Minimal risk	Minimal risk
Moldova	Moderate risk	High risk	High risk	High risk	High risk
Monaco	High risk	High risk	High risk	High risk	High risk
Mongolia	Minimal risk	Minimal risk	Minimal risk	Minimal risk	Minimal risk
Montenegro	Moderate risk	High risk	High risk	High risk	High risk
Morocco	Moderate risk	Moderate risk	Moderate risk	Moderate risk	Moderate risk
Mozambique	Low risk	Low risk	Minimal risk	Minimal risk	Minimal risk
Myanmar	Moderate risk	Moderate risk	Moderate risk	Low risk	Low risk
Namibia	Moderate risk	Low risk	Low risk	Minimal risk	Minimal risk
Nepal	Moderate risk	Moderate risk	Moderate risk	Moderate risk	Moderate risk
Netherlands	Moderate risk	High risk	High risk	High risk	High risk
New Caledonia	Moderate risk	Moderate risk	Moderate risk	Moderate risk	Moderate risk
New Zealand	Moderate risk	High risk	High risk	High risk	High risk
Nicaragua	Minimal risk	Minimal risk	Minimal risk	Minimal risk	Minimal risk
Niger	Minimal risk	Minimal risk	Minimal risk	Minimal risk	Minimal risk
Nigeria	Minimal risk	Minimal risk	Minimal risk	Minimal risk	Minimal risk
Niue	Low risk	Low risk	Low risk	Low risk	Low risk
North Korea	Minimal risk	Low risk	Low risk	Low risk	Moderate risk
North Macedonia	High risk	High risk	High risk	High risk	High risk
Norway	Minimal risk	Minimal risk	Minimal risk	Low risk	Moderate risk
Oman	Minimal risk	Minimal risk	Minimal risk	Minimal risk	Minimal risk
Pakistan	Moderate risk	Moderate risk	Moderate risk	Low risk	Low risk
Palau	Minimal risk	Minimal risk	Minimal risk	Minimal risk	Minimal risk
Palestinian Territory	Moderate risk	Moderate risk	Moderate risk	Moderate risk	Moderate risk
Panama	Minimal risk	Minimal risk	Minimal risk	Minimal risk	Minimal risk
Papua New Guinea	Minimal risk	Minimal risk	Minimal risk	Minimal risk	Minimal risk
Paraguay	High risk	Moderate risk	Moderate risk	Moderate risk	Moderate risk
Peru	Minimal risk	Minimal risk	Minimal risk	Minimal risk	Minimal risk
Philippines	Minimal risk	Minimal risk	Minimal risk	Minimal risk	Minimal risk
Poland	Moderate risk	High risk	High risk	High risk	High risk
Portugal	High risk	High risk	High risk	High risk	High risk
Puerto Rico	Moderate risk	Low risk	Low risk	Low risk	Low risk
Qatar	Minimal risk	Minimal risk	Minimal risk	Minimal risk	Minimal risk
Réunion	Moderate risk	Moderate risk	Moderate risk	Moderate risk	Moderate risk
Romania	Moderate risk	High risk	High risk	High risk	High risk
Russian Federation	Minimal risk*	Minimal risk*	Minimal risk*	Minimal risk*	Low risk*
Rwanda	Minimal risk	Minimal risk	Minimal risk	Minimal risk	Minimal risk
Saint Lucia	Minimal risk	Minimal risk	Minimal risk	Minimal risk	Minimal risk
Saint Vincent and the Grenadines	Minimal risk	Minimal risk	Minimal risk	Minimal risk	Minimal risk
Samoa	Minimal risk	Low risk	Low risk	Low risk	Minimal risk
San Marino	High risk	High risk	High risk	High risk	High risk
Sao Tome and Principe	Minimal risk	Low risk	Minimal risk	Minimal risk	Minimal risk
Saudi Arabia	Low risk	Minimal risk	Minimal risk	Minimal risk	Minimal risk
Senegal	Minimal risk	Minimal risk	Minimal risk	Minimal risk	Minimal risk
Serbia	High risk	High risk	High risk	High risk	High risk
Seychelles	Minimal risk	Minimal risk	Minimal risk	Minimal risk	Minimal risk
Sierra Leone	Minimal risk	Minimal risk	Minimal risk	Minimal risk	Minimal risk
Singapore	Minimal risk	Minimal risk	Minimal risk	Minimal risk	Minimal risk
Slovakia	Moderate risk	High risk	High risk	High risk	High risk
Slovenia	Moderate risk	High risk	High risk	High risk	High risk
Solomon Islands	Minimal risk	Minimal risk	Minimal risk	Minimal risk	Minimal risk
Somalia	Minimal risk	Minimal risk	Minimal risk	Minimal risk	Minimal risk
South Africa	High risk	High risk	High risk	Moderate risk	Moderate risk
South Georgia and South Sandwich Islands	Minimal risk	Minimal risk	Minimal risk	Minimal risk	Minimal risk
South Korea	Moderate risk	High risk	High risk	High risk	High risk
South Sudan	Minimal risk	Minimal risk	Minimal risk	Minimal risk	Minimal risk
Spain	High risk	High risk	High risk	High risk	High risk
Sri Lanka	Minimal risk	Minimal risk	Minimal risk	Minimal risk	Minimal risk
Sudan	Minimal risk	Minimal risk	Minimal risk	Minimal risk	Minimal risk
Suriname	Minimal risk	Minimal risk	Minimal risk	Minimal risk	Minimal risk
Svalbard	Minimal risk	Minimal risk	Minimal risk	Minimal risk	Minimal risk
Sweden	Minimal risk	Low risk	Moderate risk	Moderate risk	Moderate risk
Switzerland	Low risk	Moderate risk	High risk	High risk	High risk
Syria	Moderate risk	Moderate risk	Moderate risk	Moderate risk	Moderate risk
Tajikistan	Low risk	Moderate risk	Moderate risk	Moderate risk	Moderate risk
Tanzania	Minimal risk	Minimal risk	Minimal risk	Minimal risk	Minimal risk
Thailand	Low risk	Minimal risk	Minimal risk	Minimal risk	Minimal risk
Timor‐Leste	Minimal risk	Minimal risk	Minimal risk	Minimal risk	Minimal risk
Togo	Minimal risk	Minimal risk	Minimal risk	Minimal risk	Minimal risk
Trinidad and Tobago	Minimal risk	Minimal risk	Minimal risk	Minimal risk	Minimal risk
Tunisia	High risk	High risk	Moderate risk	Moderate risk	Moderate risk
Turkey	High risk	High risk	High risk	High risk	High risk
Turkmenistan	Moderate risk	Moderate risk	Moderate risk	Moderate risk	Moderate risk
Turks and Caicos Islands	Low risk	Low risk	Low risk	Minimal risk	Minimal risk
Uganda	Minimal risk	Minimal risk	Minimal risk	Minimal risk	Minimal risk
Ukraine	Moderate risk	High risk	High risk	High risk	High risk
United Arab Emirates	Minimal risk	Minimal risk	Minimal risk	Minimal risk	Minimal risk
United Kingdom	Low risk	Moderate risk	High risk	High risk	High risk
United States	Moderate risk	High risk	High risk	High risk	High risk
Uruguay	High risk	High risk	High risk	High risk	High risk
US Virgin Islands	Minimal risk	Low risk	Low risk	Minimal risk	Minimal risk
Uzbekistan	Low risk	Moderate risk	Moderate risk	Moderate risk	Moderate risk
Vanuatu	Low risk	Low risk	Low risk	Low risk	Low risk
Venezuela	Minimal risk	Minimal risk	Minimal risk	Minimal risk	Minimal risk
Vietnam	Moderate risk	Moderate risk	Moderate risk	Moderate risk	Moderate risk
Yemen	Low risk	Minimal risk	Minimal risk	Minimal risk	Minimal risk
Zambia	Moderate risk	Low risk	Low risk	Minimal risk	Minimal risk
Zimbabwe	Moderate risk	Moderate risk	Low risk	Minimal risk	Minimal risk

## APPENDIX 9

### Model evaluation

**TABLE A3:** AUC values of the consensus model and the five considered single models (combined species approach)

Model	AUC
Consensus model	0.992
GLM	0.974
GBM	0.985
GAM	0.979
ANN	0.959
FDA	0.960
RF	1.000
